# A Medical Writer Who Missed His Vocation

**Published:** 1916-09-30

**Authors:** 


### OLIVER WENDELL HOLMES.

### A Medical Writer who Missed his Vocation.

Among the medical poets Oliver Wendell Holmes has a recognised place, though he was not a poet at all, but a versatile prose writer with a most irritating habit of dropping into rhyme. Though he published several volumes of verse, all Dr. Holmes' best pieces, a mere handful, occur, characteristically enough, in his well-known essays, "The Autocrat," "The Professor," and "The Poet" "at the Breakfast Table." Without any subtlety of ear, or instinct for the fine shades of rhythm and cadence, he had a distinct capacity for setting commonplace ideas and descriptions to the regular beat of a hard mechanical metre. The stress always falls on the same place, with the wearisome repetition of a steam-hammer; there is "go" but no variety, and a rather vulgar sense of humour took the place of the rhetoric used in Macaulay's verses to which class Dr. Holmes' can be most conveniently compared. Unfortunately, in his day, the possibilities of comic opera had neither been discovered nor developed, but Wendell Holmes' real powers might have found a capital medium for their exercise if only he had had the chance of writing the libretto of a modern musical comedy. If anyone doubts the justice of this view, let him turn to the following quotations:

I saw him once before,
As he passed by the door,
And again
The pavement stones resound
As he totters o'er the ground
With his cane.

That has the hard, regular beat, the sharpness of impression, the obviousness of a good libretto; but its virtues have nothing to do with poetry, for the verse adds nothing, communicates no atmosphere, to the idea. It is obviously the recreation of a capable prose writer. The above metre reminds us of some of Campbell's, and of Campbell himself a distinguished critic has written: "Campbell had precisely that mastery of the obvious which makes rememberable lines, such as 'Distance lends enchantment to the view,' or 'Coming events cast their shadows before.' . . . They contain no poetic suggestion, they are no vital form of poetic speech; but they make statements to which verse lends a certain emphasis by its limiting form or enclosure." In the following, dreadful as it is, we catch an early glimpse of his humour. This made his prose often witty, but it hopelessly vulgarised his verse:

Thou art a female Katydid!
I know it by the trill
That quivers through thy piercing notes,
So petulant and shrill;
I think there is a knot of you
Beneath the hollow tree—
A knot of spinster Katydids—
Do Katydids drink tea?

That any writer with the smallest sense of decency or humour (the two are the same) could have printed such a verse, with such an ending, is almost incredible. But Holmes was utterly shameless in this way, and his most shameless exercises are contained in the poems which, like that quoted, deal with some scientific subject, medical instrument, or technical term. People, however, are accustomed to pretend to enjoy these vulgarities, and there are several after-dinner verses by him, apparently written by request. Here is a sample, followed by a verse from his poem on the "Stethoscope," then a new toy, laughed at by the more conservative practitioners. In "Verses for After Dinner" Dr. Holmes apostrophises the president of the Φ B K Society thus:

While others enlarge on the boiled and the roast,
He serves a raw clergyman up with a toast,
Or catches some doctor quite tender and young,
And basely insists on a bit of his tongue.

In the "Stethoscope Song" he ridicules the young up-to-date practitioner who mistakes the sound of a bee's buzzing in his new instrument for "amphoric buzzing" on one occasion, and *bruit de râpe* on another. The obvious moral is drawn in the last verse:

Now use your ears, all you that can,
But don't forget to mind your eyes,
Or you may be cheated, like this young man,
By a couple of silly, abnormal flies.

It is pleasant to turn from this stuff to two examples of his better verse. The first is his most-admired poem, "The Chambered Nautilus," in which he describes the habit of this small cephalopod in building a series of chambers as it grows to maturity. The last verse may be given in full for the benefit of any reader who is not already acquainted with it.

Thanks for the heavenly message brought by thee,
Child of the wandering sea,
Cast from her lap, forlorn!
From thy dead lips a clearer note is born
Than ever Triton blew from wreathèd horn!
While in mine ear it rings,
Through the deep caves of thought I hear a voice that sings:
Build thee more stately mansions, O my soul,
As the swift seasons roll!
Leave thy low-vaulted past!
Let each new temple, nobler than the last,
Shut thee from heaven with a dome more vast,
Till thou at length art free,
Leaving thine outgrown shell by life's unresting sea!

That has poetical substance, but even here the dragging in of Wordsworth's famous phrase reveals a slackening of imagination which shows that Holmes could never long rely on his own wings. In another of his poems, "Homesick in Heaven," two lines occur which indicate just how far his tethered pen could reach:

The flower once opened may not bud again,
The fruit once fallen finds its stem no more.

It is far from first-rate, but it was the best that Holmes could do in a medium that he should never have attempted at a time when the proper use for such vitality of presentment and rhyming faculty as he had had not been discovered.

The moment we turn to his prose, however, we are in a different atmosphere altogether. Prose has vitality, but poetry has wings; and the first paper in his "Autocrat of the Breakfast Table," which was published in 1857, is enough to prove his quality. What could be better than this? "It is a good sign for one to have his feet grow cold when he is writing"; or this, "Talk about conceit as much as you like, it is to human character what salt is to the ocean; it keeps it sweet and renders it endurable. When one has *all* the conceit taken out of him . . . he will fly no more." Again, "I will tell you what I have found spoil more good talks than anything else: long arguments on special points between people who differ on fundamental principles." Again, "I should say that the most frequent work of a logical mind was to build a *pons asinorum* over chasms which shrewd people can bestride without such a structure." This last is exactly what Bergson has been teaching us. These and many other witty sayings no doubt found their way into Holmes' pages from his habit of making notes. The born author's instinct is infallibly shown in this aphorism: "It is a capital plan to carry a tablet with you, and, when you find yourself felicitous, take notes of your own conversation." Hamlet in exclaiming "My tablets! meet it is I set it down" and Sir Hugh Evans in the *Merry Wives* in declaring "I must make a prief of it in my note-book," betray the writer's instinct to snap up unconsidered trifles. Bad writers spin their stuff out of their heads; but the men who make us laugh are the fine observers. And, since memory is treacherous, the painter's sketch-book and the author's note-book have to be pulled out by their owners wherever they go.

Of Holmes' novels (including "Elsie Venner," which has been called the "snake story of literature"), of his "Lives" of Motley and Emerson, of his two visits to Europe, there is no space to speak. But the fact that he wrote them proves his versatility, and versatility is precisely the quality which is most useful to the essay writer who, like Holmes, would follow in the classic steps of Addison, Hazlitt, and Leigh Hunt. Versatility is also one of the qualities which go to make a good librettist.

His life is not remarkable for incident. Born in 1809 at Cambridge, Massachusetts, he began to practise medicine at Boston in 1835, became Professor of Anatomy at Dartmouth College three years later, and finally settled down in the Chair of Anatomy and Physiology at Harvard in 1847, a post which he held for thirty-five years. His person is described as that of a man with white, straight hair, waving from left to right, dark eyes, a large mobile mouth, long upper lip, and loose cheeks. These are the features of a born busybody, speculator, and humorist, who, while an ornament of the medical profession, missed his literary destiny, which should have been to write for America such librettos of humour and fancy as Gilbert in a subtler and more musical vein so delightfully gave us over here.

